# A harm-reduction model of abortion counseling about misoprostol use in Peru with telephone and in-person follow-up: A cohort study

**DOI:** 10.1371/journal.pone.0189195

**Published:** 2018-01-10

**Authors:** Daniel Grossman, Sarah E. Baum, Denitza Andjelic, Carrie Tatum, Guadalupe Torres, Liza Fuentes, Jennifer Friedman

**Affiliations:** 1 Advancing New Standards in Reproductive Health (ANSIRH), Bixby Center for Global Reproductive Health, Department of Obstetrics, Gynecology and Reproductive Sciences, University of California San Francisco, Oakland, California, United States of America; 2 Ibis Reproductive Health, Oakland, California, United States of America; 3 International Planned Parenthood Federation, Western Hemisphere Region, New York City, New York, United States of America; 4 Instituto Peruano de Paternidad Responsable (INPPARES), Lima, Peru; National Institute of Health, ITALY

## Abstract

**Background:**

In Peru, abortion is legal only to preserve the life and health of the woman. A non-profit clinic system in Peru implemented a harm-reduction model for women with unwanted pregnancy that included pre-abortion care with instructions about misoprostol use and post-abortion care; they started offering telephone follow-up for clients in 2011. This study aimed to evaluate the effectiveness and safety of the harm-reduction model, and to compare outcomes by type of follow-up obtained.

**Methods:**

Between January 2012 and March 2013, 500 adult women seeking harm-reduction services were recruited into the study. Telephone surveys were conducted approximately four weeks after their initial harm-reduction counseling session with 262 women (response rate 52%); 9 participants were excluded. The survey focused on whether women pursued an abortion, and if so, what their experience was. Demographic and clinical data were also extracted from clinic records.

**Results:**

Eighty-six percent of participants took misoprostol; among those taking misoprostol, 89% reported a complete abortion at the time of the survey. Twenty-two percent obtained an aspiration after taking misoprostol and 8% self-reported adverse events including hemorrhage without transfusion, infection, or severe pain. Among women who took misoprostol, 46% reported receiving in-person follow-up (in some cases both telephone and in-person), 34% received telephone only, and 20% did not report receiving any form of follow-up. Those who had in-person follow-up with the counselor were most likely to report a complete abortion (<0.001). Satisfaction with both types of follow-up was very high, with 81%-89% reporting being very satisfied.

**Conclusions:**

Liberalization of restrictive abortion laws is associated with improvements in health outcomes, but the process of legal reform is often lengthy. In the interim, giving women information about evidence-based regimens of misoprostol, as well as offering a range of follow-up options to ensure high quality post-abortion care, may reduce the risks associated with unsafe abortion.

## Introduction

Abortion is legally restricted throughout most of Latin America, leading many women with unwanted pregnancies to turn to clandestine or unsafe providers, or to attempt self-induction of abortion using a variety of techniques, some of which may be unsafe or ineffective. The most recent estimate of the annual incidence of abortion in South America is 47 per 1,000 women aged 15–44; this rate has been stable since 1990 and is higher than the global abortion rate of 35 per 1,000 women [1]. Throughout Latin America, unsafe abortion is responsible for 10–20% of maternal deaths [2]. In Peru, where abortion is legal only to preserve the life and health of the woman, a large, population-based survey of women aged 18–29 found that 11.6% reported a prior induced abortion [3]. Liberalizing abortion laws to allow for provision of safe, clinic-based abortion care could lead to a reduction in abortion-related morbidity and mortality in the region.

In settings where mifepristone is not available, the World Health Organization recommends a regimen of misoprostol alone in a dosage of 800 mcg administered vaginally or sublingually, repeated up to three times at intervals of 3–12 hours to induce an abortion [4]. Misoprostol is a safer and more effective alternative to other forms of abortion self-induction [5], and studies have documented widespread availability of the drug in Latin America [6–9]. Two modeling studies have demonstrated that replacement of unsafe abortion methods with misoprostol could significantly improve health outcomes and reduce costs associated with treating complications of unsafe abortion in Latin America [10, 11]. The decline in the rate of serious complications from unsafe abortion in Peru has been attributed to misoprostol availability in the country [12], although studies have also demonstrated that pharmacy staff in Latin America provide limited information about appropriate regimens of misoprostol [13, 14].

A risk- or harm-reduction approach to unsafe abortion has shown promise in Uruguay. In response to high maternal mortality from unsafe abortion, the non-governmental organization Iniciativas Sanitarias developed a program to ensure that women planning to have an abortion outside of the mainstream health system have adequate counseling and care before and after the procedure. The aim of this harm-reduction approach is to decrease the negative health effects of unsafe abortion among particularly vulnerable populations [15]. The model asserts that even in contexts where abortion is legally restricted, it is legal and appropriate for health professionals to provide information and medical care to women before and after an illegal abortion [15].

The Uruguay model, which was implemented before the law was changed in 2012 to allow abortion on request in the first trimester, includes informing women with unintended pregnancies and no legal grounds for clinic-based induced abortion about the risks involved in clandestine pregnancy termination, according to gestational age and method used. In particular, women are given information about the evidence indicating that appropriate doses of misoprostol early in pregnancy may safely and effectively terminate an early pregnancy. The objectives of the post-abortion follow-up visit are to identify medical complications and ongoing pregnancy, as well as to provide emotional support and post-abortion contraception as needed. Evaluation of the initial implementation of the program at the main maternity hospital in Uruguay demonstrated that complications and deaths from unsafe abortion decreased during the study period compared to previous years [15]. The evaluation also found that a high proportion of women used misoprostol and returned for the follow-up visit, indicating that women were receptive to the program [15].

A similar harm-reduction program was implemented at the Instituto Peruano de Paternidad Responsable (INPPARES), a non-governmental sexual and reproductive health organization that provides educational and clinical services for women and men in Peru. INPPARES implemented the harm-reduction model in its largest clinic in Lima in 2006. The model has since expanded to various clinic sites in Lima as well as a high-volume clinic in Chimbote, a small town along the northwestern coast of Peru. Similar to the model in Uruguay, women at INPPARES have an initial consultation with a trained counselor that includes information about safe and unsafe methods of self-induction. Misoprostol is presented as a safer alternative to other methods, and women are given information on appropriate dosing, side effects, and effectiveness. The model encourages women to attend a follow-up visit, either for antenatal or post-abortion care depending on their decision. In July-December 2011, INPPARES reported that 30% of women seeking harm-reduction services returned for an in-person follow-up visit.

In November 2011, INPPARES started offering follow-up services by telephone in addition to in-person visits to women who received harm-reduction counseling. The option of telephone follow-up was added to make the process more user-friendly for women with the hope that it would increase the proportion of women obtaining follow-up care. The objectives of this study were to document women’s experiences with and acceptability of this harm-reduction model of care, to document the effectiveness and safety of the harm-reduction model, and to compare outcomes between women who receive telephone vs. in-person follow-up. Results from this study have the potential to provide evidence for scale-up of the harm-reduction model and telephone follow-up services in Peru as well as other countries where abortion is legally restricted.

## Materials and methods

Between January 2012 and March 2013, women who were eligible for harm-reduction services based on the clinic standard of practice at INPPARES clinic study sites in Lima (2 sites) or Chimbote (1 site) were recruited for the study. Women who spoke Spanish and were at least 18 years old were eligible to participate in the study.

Women went through the standard harm-reduction consultation, which included confirmation of pregnancy, assessment of gestational age by ultrasound and counseling about pregnancy options, including the use of misoprostol for those opting to terminate, and any other needed emotional support. At the end of the initial harm-reduction consultation, clinic counselors described in-person and telephone follow-up options to all women. If a woman wanted the counselor to call her, this call was scheduled in advance, although women were also encouraged to call the clinic at any point if they had questions. The counselor then introduced the research study to potential participants, and if the woman was interested in participating in the study, the counselor reviewed the informed consent form with her. When a woman agreed to participate in the study, she orally consented, and a copy of the form was given to her. Women who gave informed consent were asked to provide a telephone number where they could be contacted and a name or pseudonym they would like to be identified by when the interviewer called. Alternatively, if a woman preferred to call, she was given a card with the name and telephone number of the interviewer, the approximate date when she should call, and her study ID number.

Women who chose telephone follow-up underwent an assessment that was based on a study in the US [16]. Telephone assessment occurred approximately one week after counseling, and clinic staff asked whether women had taken misoprostol. Those indicating they had taken misoprostol were asked a series of questions to confirm successful completion of the abortion and to gather information about the woman’s experience. The questions used to evaluate abortion completion were:

1. Did you have pain and bleeding heavier than a normal period?
2. Did you expel blood clots and/or tissue?
3. Is your bleeding currently less than the heaviest day of your period?
4. Have the symptoms of pregnancy (nausea, swollen breasts) disappeared?

If the answer was “no” or “not sure” to any of these, counselors encouraged women to come to the clinic. Women were given information about additional assessments that could be done, such as ultrasound or urine pregnancy testing, to confirm completion of the abortion. Counselors also provided emotional support and information about contraception at the time of the follow-up call.

Data for this analysis came from two sources: 1) participants were called by study interviewers approximately one month after recruitment to complete a questionnaire via telephone (which was separate from any telephone follow-up provided by clinic staff) and 2) we abstracted demographic and clinical data from clinic records at the initial counseling visit. Clinical data for enrolled participants were obtained from information normally documented in patient records as a part of the clients’ regular care.

The telephone questionnaire aimed to examine women’s experiences with and acceptability of the harm-reduction model of service provision. The questionnaire included closed- and open-ended items asking women about their pregnancy decision and outcome, and for women who chose to have an abortion, experiences obtaining and using misoprostol, the amount and timing of medication taken, and experiences with side effects and complications. Women were asked about their experience and satisfaction with the initial and follow-up sessions and reasons for not returning for follow-up. If women scheduled three calls with the interviewer without successfully completing the interview or were unreachable three months after their initial consult, they were considered lost to follow-up. Participants were reimbursed approximately USD $7 in cellular phone time or cash for completing the telephone questionnaire.

Clinical data were extracted from patient charts by Investigación en Salud y Demografía (INSAD), a non-profit health and demography research group in Mexico. Clinical data were collected on paper forms for all women who obtained harm-reduction services and these forms were sent to INSAD for data entry. INSAD extracted the clinical data of study participants based on their patient IDs.

Data from telephone questionnaires were captured in the encrypted online survey program, SurveyMonkey (SurveyMonkey Inc.,Palo Alto, CA), either while the interview was conducted or afterward if internet was unavailable. Questionnaire data, as well as demographic and clinical information, were entered into a database with no personal identifiers and analyzed in StataIC 12 (StataCorp LP, College Station, TX). The study was approved by the Allendale Investigational Review Board which is the IRB usually used by Ibis Reproductive Health (where the principal investigator was based at the time of data collection). INPPARES leadership reviewed the protocol internally and relied on the Allendale IRB approval.

In order to compare women across type of follow-up and evaluate telephone follow-up specifically, we categorized participants into three groups based on the type of follow-up they received: telephone only, in-person (including in-person only, or both in-person and telephone follow-up), and no follow-up. Some women with telephone-only or no follow-up did report having an ultrasound performed at a facility other than INPPARES to confirm the abortion was complete; however, if the woman did not self-report this clinical encounter as in-person follow-up, it was not categorized as such during analysis. Commonly women did not tell the provider who performed the ultrasound that they had had an abortion.

In order to assess whether women took the misoprostol regimen recommended by INPPARES, we asked women about how many pills they took at each dosing and the amount of time that passed between doses. Women were considered to be *within* the medically-recommended range if they took two or three doses of 800 mcg of misoprostol per dose and *outside* the medically-recommended range if they 1) took less than a total dose of 1600 mcg, 2) took a total dose of 1600–2400 mcg but in a number of doses that did not align with recommendations, 3) took more than 2400 mcg regardless of number of doses.

The sample size for this study was based on feasibility and determined by the number of participants that could be recruited and followed up during the 15-month data collection period. We used descriptive statistics to describe the study sample and identify the clinical trajectories of patients, including the proportion that chose to have an abortion, misoprostol use, the regimen they used, and clinical outcomes. Satisfaction with initial and follow-up visits and selected clinical outcomes were compared across follow-up categories using Fisher’s exact tests and t-tests.

## Results

**Fig 1:**
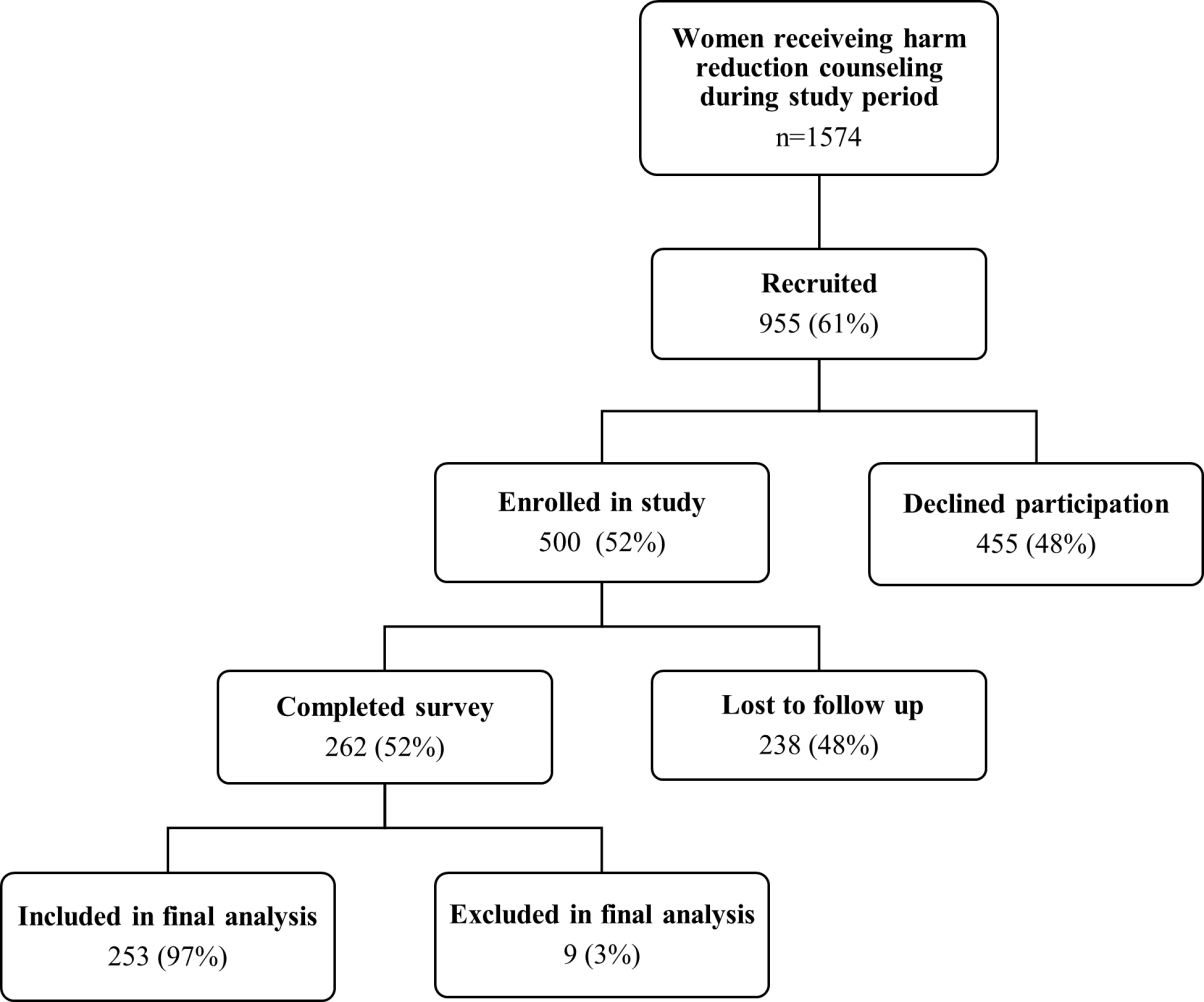
Study sample. The figure shows the flow of patients through the study.

There were 1,574 women who received harm-reduction counseling at INPPARES study sites during the study period. Clinic counselors invited 955 (61%) of these women to participate in the evaluation, and a total of 500 women (52% of women recruited) agreed to participate and were enrolled in the study. Among those that enrolled, 48% were lost to follow-up. Nine women that completed the survey (3%) were excluded from analysis: five did not meet inclusion criteria (they attended counseling for contraception options, they did not have an unwanted pregnancy, or they were younger than 18 years of age), and four answered fewer than 30% of the questions. Therefore, 253 women were included in analysis (Fig 1).

Demographic information from abstracted clinic forms was available for 229 participants completing the interview and is summarized in Table 1. The women in the sample had a mean age of 26 (range: 18–47 years). The majority had completed secondary education (69%) and were single (71%). Almost half of the women (46%) did not report any paid work, while 31% had formal paid work and 23% had informal paid work. The majority of participants had never given birth (62%) and reported no prior abortions (73%). The mean gestational age at ultrasound was 6 weeks (range: 4–10 weeks). Demographic data were also available for 230 women who consented for the study but were unable to be contacted for the follow-up interview. Those lost to follow-up were similar to those who completed the interview, except more married women and more women in Chimbote were lost to follow-up; there was also a trend toward more women without paid work being lost to follow-up (Table 1).

**Table 1:** Participant characteristics.

		Participants who completed survey	Participants lost to follow up	p-value^c^
		(n = 253)^a^	(n = 238)^b^	
		n (%)	n (%)	
**Recruitment City**				<0.01
	Lima	137 (54.1)	105 (44.1)	
	Chimbote	92 (36.4)	125 (52.5)	
	Missing	24 (9.5)	8 (3.4)	
**Age**				
	Mean	26	26.5	
	Median	24	24	
	18–25	116 (45.8)	116 (48.7)	0.36
	26–30	48 (19.0)	40 (16.8)	
	31–35	24 (9.5)	36 (15.1)	
	>35	23 (9.0)	21 (8.8)	
	Missing	42 (16.6)	25 (10.5)	
**Education**				0.559
	Incomplete secondary	9 (3.6)	14 (5.9)	
	Complete secondary	159 (62.8)	158 (66.4)	
	University	61 (24.1)	58 (24.3)	
	Missing	24 (9.5)	8 (3.4)	
**Relationship Status**				<0.05
	Single	162 (64.0)	148 (62.2)	
	Partnered or living together	44 (17.4)	48 (20.1)	
	Married	11 (4.3)	27 (11.3)	
	Divorced/Separated/Widowed	12 (4.7)	7 (3.0)	
	Missing	24 (9.5)	8 (3.4)	
**Paid work**				0.051
	Informal	53 (20.9)	38 (16.0)	
	Formal	70 (27.7)	61 (25.6)	
	None	105 (41.5)	131 (55.0)	
	Missing	25 (9.9)	8 (3.4)	
**Parity**				
	Mean	0.66	0.73	
	Median	0	0	
	0	142 (56.1)	136 (57.1)	0.599
	1	44 (17.4)	42 (17.6)	
	≥2	43 (17.0)	52 (21.8)	
	Missing	24 (9.5)	8 (3.4)	
**Prior abortion**				0.354
	0	168 (66.4)	158 (66.4)	
	1	45 (17.8)	58 (24.4)	
	≥2	16 (6.3)	14 (5.9)	
	Missing	24 (9.5)	8 (3.4)	
**Gestational age (weeks)**				
	Mean	5.8	5.9	
	Median	6	6	
	≤6 weeks	174 (68.8)	165 (69.3)	0.427
	7–8 weeks	38 (15.0)	47 (19.7)	
	9–10 weeks	10 (4.0)	7 (2.9)	
	Missing	31 (12.3)	19 (8.0)	

^a^24 clinic records were unavailable.

^b^8 clinic records were unavailable.

^c^p-values are calculated without missing values.

### Harm-reduction initial counseling and follow-up

**Fig 2:**
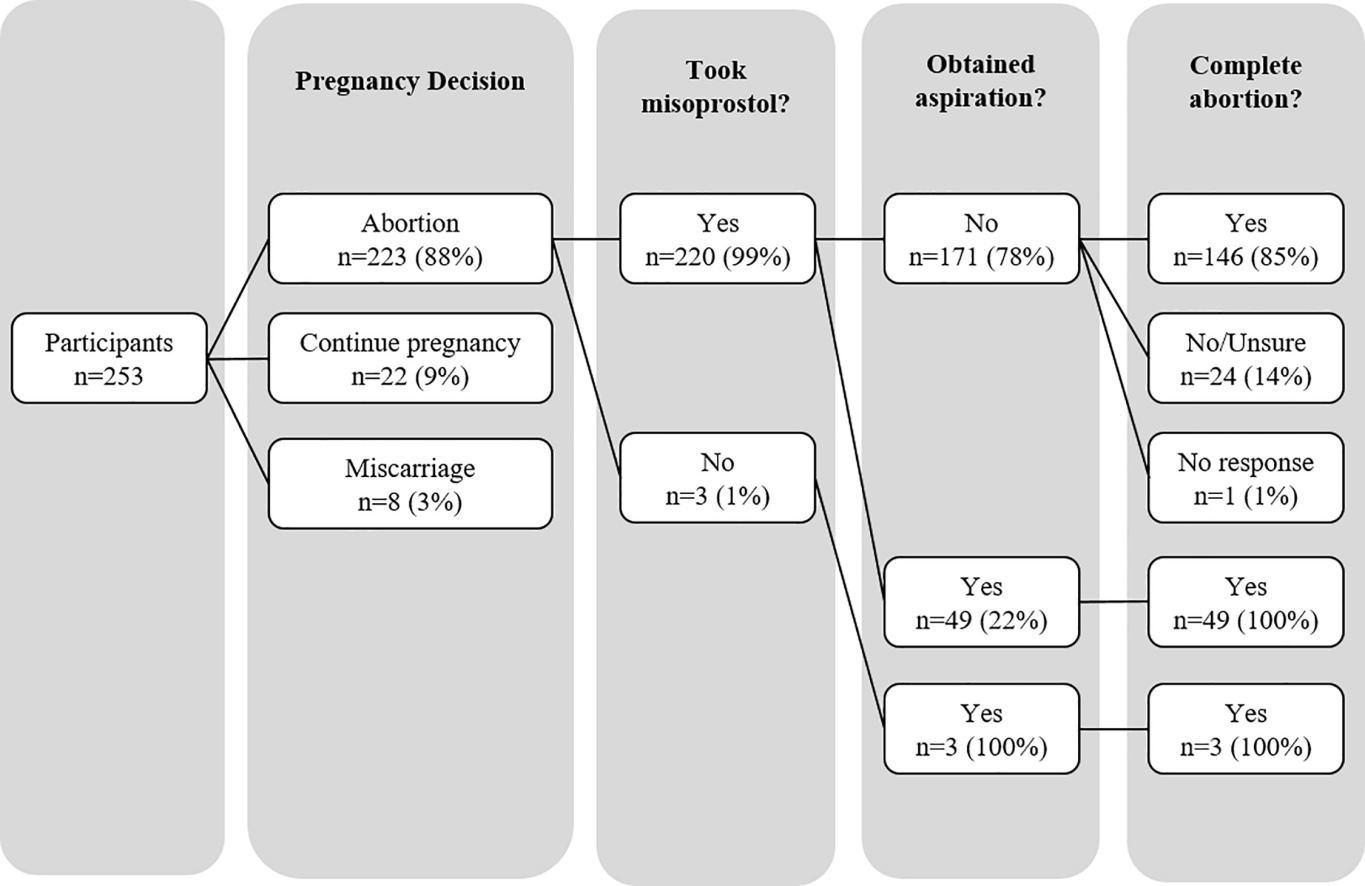
Women’s clinical trajectories. The number and proportion of study participants who had an abortion or miscarriage or continued the pregnancy are shown. For those who had an abortion, the number and proportion who reported taking misoprostol, obtaining a uterine aspiration and having a complete abortion by the time of the study interview are shown.

The vast majority of participants (85%) reported being very satisfied with their initial harm-reduction consultation visit. Most women reported that the information was very easy (57%) or somewhat easy (29%) to understand, and the vast majority said they felt comfortable asking questions (97%), although a significantly lower proportion of those with no follow-up (88%) reported feeling comfortable asking questions (Table 2). Most women reported they decided to have an abortion (88%), and of those, all took misoprostol except three women who obtained a surgical termination. A small proportion of women (3%) reported they had a miscarriage, and 9% decided to continue the pregnancy (Fig 2). Among the 220 women who took misoprostol, 46% reported receiving in-person follow-up (12% received in-person only and 34% received both telephone and in-person), 34% received telephone only, and 20% did not receive any form of follow-up. Of the participants reporting no follow-up (n = 43), the most common reason was that they did not have time or had other personal commitments (n = 30). Of note, women recruited in Chimbote, the smaller, more rural recruitment site, were more likely than those recruited in Lima to use telephone-only follow-up (49% vs. 24%, p<0.001) and have no follow-up (36% vs 8%, p<0.001).

**Table 2:** Satisfaction and acceptability of harm-reduction initial consult and follow-up services among women who used misoprostol.

		All participants who took misoprostol	Telephone only follow-up	Any in-person follow-up^a^	No follow-up	p-value
		[n = 220]	[n = 75]	[n = 102]	[n = 43]	
		n (%)	n (%)	n (%)	n (%)	
**Recruitment city**						<0.001
	Lima	132 (60.0)	32 (42.7)	89 (87.3)	11 (25.6)	
	Chimbote	88 (40.0)	43 (57.3)	13 (12.7)	32 (74.4)	
**Satisfaction with initial consult**		** **				0.966
	Very satisfied	187 (85.0)	64 (85.3)	87 (85.3)	36 (83.7)	
	Somewhat satisfied or unsatisfied	33 (15.0)	11 (14.7)	15 (14.7)	7 (16.3)	
**Ease in understanding information received about misoprostol at initial consult**						0.710
	Very easy	126 (57.3)	38 (50.7)	61 (59.8)	27 (62.8)	
	Somewhat easy	63 (28.6)	24 (32.0)	28 (27.5)	11 (25.6)	
	Very or somewhat hard	19 (8.6)	9 (12.0)	7 (6.9)	3 (7.0)	
	Reported not receiving information on misoprostol	6 (2.7)	2 (2.7)	2 (2.0)	2 (4.7)	
**Felt comfortable asking questions at initial consult**		213 (96.8)	73 (97.3)	102 (100.0)	38 (88.4)	0.001
**Satisfaction with telephone follow-up**						
	Very satisfied		61 (81.3)	71 (88.8)	-	0.352
	Somewhat satisfied or unsatisfied		12 (16)	9 (11.3)	-	
**Satisfaction with in-person follow-up**						
	Very satisfied		-	83 (81.4)	-	
	Somewhat satisfied or unsatisfied		-	10 (9.8)	-	
**Felt comfortable asking question of counselor at follow-up **			71 (94.7)	92 (90.2)	-	1.000
**Would recommend service to a friend**		215 (97.7)	75 (100)	101 (99.0)	39 (90.7)	0.015
**Would use service again if necessary**		210 (95.5)	75 (100)	97 (95.0)	38 (88.4)	0.031
**Interested in text message follow-up**						0.057
	Very or somewhat interested	170 (77.3)	57 (76.0)	74 (72.6)	39 (90.7)	
	Not interested	48 (21.8)	17 (22.7)	28 (27.5)	3 (7.0)	

Column numbers do not sum to total because of missing data.

^a^74% of these participants had telephone follow-up in addition to in-person follow-up.

Overall, satisfaction with both types of follow-up was very high, with 81%-89% reporting being very satisfied. Women who did not have follow-up were less likely to say they would recommend the service to a friend compared to those with any type of follow-up (91% vs. 100% for telephone only follow-up and 99% for any in-person follow-up, p = 0.02). Similarly, a lower proportion of women who had no follow-up said they would use the service again if they were in a similar situation (88% vs. 100% for telephone only follow-up and 95% for any in-person follow-up, p = 0.03) (Table 2).

As an alternative to both telephone and in-person follow-up, we asked women if they would be interested in a program that provided text message follow-up. Approximately three-quarters (77%) of participants were very or somewhat interested in the text message option, with a trend toward higher interest among women who did not have follow-up as compared to those with telephone only or in-person (Table 2).

### Misoprostol use and clinical outcomes

All study participants who reported obtaining misoprostol also reported taking the medication to self-induce an abortion (n = 220). In addition to the information received from the counselor, women reported getting information from other sources, including family and friends and the internet (Table 3). Women reported traveling a median of 30 minutes from their home to obtain the medication at pharmacies (80%), from family or friends (11%) or from the internet (4%). Forty-six percent found it somewhat or very easy to get misoprostol, 37% found it somewhat hard, and 14% found it very hard. The most common challenge was that the pharmacy refused to sell the medication or required a doctor’s prescription. The average cost of a single misoprostol tablet was approximately $3, and all but 10 women were able to obtain the amount recommended during their harm-reduction consultation visit (data not shown).

**Table 3:** Experience obtaining and taking misoprostol.

		All participants who took misoprostol
		(n = 220)
		n (%)
**Information about misoprostol from sources other than initial consult**^**a**^		
	Pharmacy	8 (3.6)
	Friends/Family	34 (15.5)
	Internet	37 (16.8)
	Provider outside of INPPARES	5 (2.3)
	Other	4 (1.8)
**Location where misoprostol obtained**^**a**^		
	Pharmacy	175 (79.6)
	Friends/Family	23 (10.5)
	Internet	9 (4.1)
	Other	13 (5.9)
**Distance traveled from home to obtain misoprostol**		
	Median [mins]	30
**Difficulty in obtaining misoprostol**		
	Very or somewhat easy	101 (45.9)
	Somewhat hard	82 (37.3)
	Very hard	31 (14.0)
**Reasons it was hard to obtain misoprostol**^**a**^		
	Pharmacy would not sell	114 (51.8)
	Misoprostol was not available at the pharmacy	16 (7.2)
	Felt embarrassed or worried	8 (3.6)
	Did not have enough money	5 (2.3)
	Pharmacy would not sell enough pills	4 (1.8)
	Difficulty finding or traveling to a pharmacy	3 (1.4)
	Other	7 (3.2)
**Degree of certainty about taking misoprostol accurately**		** **
	Very sure	82 (37.3)
	Somewhat sure	70 (31.8)
	Very or somewhat unsure	68 (30.9)
**Regimen used**		** **
	Took within the medically-recommended range	158 (71.8)
	Took outside the medically-recommended range	60 (27.3)
	Took less than medically-recommended range (<8 pills)	43 (19.6)
	Medically-recommended dosage but incorrect regimen	9 (4.1)
	Took more than medically-recommended range (>12 pills)	8 (3.6)

Column numbers do not sum to total because of missing data.

^a^Participants able to choose multiple responses when applicable.

At the time of taking misoprostol, 37% of women reported being very sure and 32% reported being somewhat sure that they knew how to take the correct regimen. Almost three-quarters of women took misoprostol according to the recommended regimen, while 27% of women followed a regimen outside the recommendations. Among those that did not follow the recommended regimen, the majority took less than 1600 mcg of misoprostol (Table 3).

Among women who took misoprostol, 8% (n = 17) reported adverse events including hemorrhage (without transfusion), infection, or severe pain. Treatments included antibiotics (2%; n = 5), intravenous fluids (1%; n = 1) and/or surgical abortion (4%; n = 8). Six women (3%) reported no treatment for their symptoms. Only two of the women (1%) spent one or more nights in a hospital, both of whom reported receiving treatment for infection. Rates of adverse events were similar across women with in-person and telephone follow-up, and importantly, no adverse events were reported among women with no follow-up (Table 4).

**Table 4:** Clinical outcomes and contraceptive use by follow-up type among women who used misoprostol.

		All participants who took misoprostol	Telephone follow-up only	Any in-person follow-up^a^	No follow-up	
		[n = 220]	[n = 75]	[n = 102]	[n = 43]	
		n (%)	n (%)	n (%)	n (%)	p-value
**Reported adverse events**		17 (7.7)	7 (9.3)	10 (9.8)	0 (0.0)	0.106
** **	Hemorrhage (without transfusion)	5 (2.3)	1 (1.3)	4 (3.9)	0 (0.0)	
** **	Infection	5 (2.3)	3 (4.0)	2 (2.0)	0 (0.0)	
** **	Severe pain	5 (2.3)	2 (2.7)	3 (2.9)	0 (0.0)	
** **	Other	2 (0.9)	1 (1.3)	1 (1.0)	0 (0.0)	
**Had surgical abortion following misoprostol use**		49 (22.3)	14 (18.7)	35 (34.3)	0 (0.0)	<0.001
**Reported complete abortion at time of survey**						<0.001
	Yes	195 (88.6)	66 (88.0)	99 (97.1)	30 (69.8)	
	No	4 (1.8)	2 (2.6)	1 (1.0)	1 (2.3)	
	Not sure	20 (9.1)	7 (9.3)	1 (1.0)	12 (27.9)	
**How participant knew abortion was complete**^**b**^						
	Ultrasound	147 (66.8)	43 (57.3)	85 (83.3)	19 (44.2)	
	Regular period	22 (10.0)	9 (12.0)	6 (5.9)	7 (16.3)	
	Bleeding stopped	13 (5.9)	5 (6.7)	5 (4.9)	3 (7.0)	
	Saw products of conception	10 (4.5)	4 (5.3)	2 (2.0)	4 (9.3)	
	Pregnancy symptoms disappeared	7 (3.2)	4 (5.3)	1 (1.0)	2 (4.7)	
	Pregnancy test	3 (1.4)	1 (1.3)	1 (1.0)	1 (2.3)	
	Counselor told her	8 (3.6)	3 (4.0)	5 (4.9)	N/A	
	Other	18 (8.1)	6 (8.0)	7 (6.9)	5 (11.6)	
**Post-abortion contraceptive use**						0.064^c^
	No method	100 (45.5)	34 (45.3)	40 (39.2)	26 (60.5)	
	Using method	120 (54.5)	41 (54.7)	62 (60.8)	17 (39.5)	
	**Type of method**^d^					
	Hormonal methods	76 (34.6)	27 (36.0)	38 (37.3)	11 (25.6)	
	Long-acting reversible methods	23 (10.5)	6 (8.0)	13 (12.7)	4 (9.3)	
	Barrier or rhythm methods	21 (9.6)	8 (10.7)	11 (10.8)	2 (4.7)	

^a^74% of these participants had telephone follow-up in addition to in-person follow-up.

^b^Participants able to choose multiple responses when applicable.

^c^p = 0.04 when women with follow-up of any kind (telephone only, in person only or both) are compared to those without follow-up (58% vs 40%, respectively).

^d^Hormonal methods include oral contraceptive pill, patch, injectable, emergency contraception; Long-acting reversible methods include IUD and implant.

Eighty-nine percent of women who took misoprostol reported having a complete abortion and when asked how they knew it was complete, the majority said they had an ultrasound (67%) (Table 4). The proportion reporting ultrasound was highest among those with in-person follow-up (80%), but it was also commonly reported among those with telephone-only (57%) or no follow-up (44%). Twenty-two percent reported having a surgical procedure such as uterine curettage or vacuum aspiration by the time of the follow-up interview, and all of these reported that the abortion was now complete. There were 24 women (11%) who took misoprostol and reported their abortion was incomplete or they did not know it if was complete at the time of the telephone interview. Women who reported being unsure if the abortion was complete said they planned to get medical attention either at INPPARES (n = 4, 16%) or another facility (n = 6, 25%), while one said she planned to take another dose of misoprostol. Those who had any in-person follow-up were most likely to report the abortion was complete (97%), followed by telephone-only (88%) and no-follow-up (70%) (Table 4).

At the time of the telephone questionnaire, 55% of women who took misoprostol were using a contraceptive method, and women with follow-up of any kind (in-person, telephone or both) were more likely to use post-abortion contraception than those without follow-up (58% vs 40%; p = 0.04). Most women using contraception reported using more effective methods, including IUD, implants, and hormonal methods such as the pill or injectable (Table 4).

## Discussion

This study is one of the few to document outcomes with a harm-reduction model of care aimed at reducing the risks associated with unsafe abortion. We found that the vast majority of women receiving the initial harm-reduction counseling—including those who did not return for follow-up—went on to take misoprostol appropriately and terminated their pregnancies in this context. Although satisfaction with the model was high, women also report challenges obtaining the drug, and some said they were unsure about how to take it. These findings suggest that other models of care that include medication provision, including expanding the provision of care within the legal guidelines or through telemedicine, could further improve access to safe abortion in settings with restrictive legislation [17] [18] [19]. In 2014, Peru launched a national protocol for legal abortion, which will help physicians to better provide services within the legal framework [20]. Ultimately, legal reform is also needed to ensure women’s health and reproductive rights are fully respected.

The clinical outcomes we observed with this model of care are similar to published data on the use of misoprostol alone for early medication abortion. Among women taking misoprostol, 22% reported having a surgical procedure to complete the abortion, which is similar to the efficacy of the misoprostol alone regimen (approximately 85%) reported in large clinical trials [21], although some women in our study (14%) were still unsure if the abortion was complete at the time of the interview. The proportion receiving a surgical procedure may be somewhat higher in this legally restricted setting given that women may seek follow-up care without giving complete information about their medical history. For example, they may seek care saying they had a spontaneous miscarriage, and physicians may be more likely to intervene with a surgical evacuation. Higher rates of curettage have been reported among women living in Latin America who access a telemedicine site providing medical abortion with mifepristone and misoprostol compared to those living in Western Europe [19].

Few women reported serious adverse events after taking misoprostol on their own. Only two of 220 women (1%) reported a major complication, defined as transfusion, abdominal surgery or overnight hospitalization [22]; both of these women reported being hospitalized for treatment of an infection. The low prevalence of major complications is similar to reported findings from the Uruguay harm-reduction model [23]. These studies corroborate data from Brazil, where serious complications were much less common with use of misoprostol compared to other methods of clandestine abortion [5]. Other reports from Peru suggest that maternal mortality related to abortion has declined with more widespread access to misoprostol [12].

We found that adding the option of telephone follow-up was associated with more women who underwent harm-reduction counseling obtaining some type of follow-up (77%) compared to historical data (30% obtained in-person follow-up in July-December 2011). At the same time, satisfaction with telephone follow-up was high and similar to that reported with in-person follow-up. Women who had telephone follow-up were somewhat less certain the abortion was complete compared to those that had in-person follow-up, but they were more certain than those who had no follow-up. Most women who had telephone follow-up and were not sure the abortion was complete reported they were planning to go back to the clinic or take another dose of misoprostol, suggesting that accurate information had been transmitted to these women. It is notable that three-quarters of the participants reported being interested in a text-message option for follow-up, and more research should be done to explore this model in Peru.

Since this research was performed, studies have documented the accuracy of a multi-level pregnancy test to identify women with ongoing pregnancy after medication abortion [24, 25]. These tests have multiple strips to detect different concentration ranges of hCG in urine, and the hCG concentration usually falls after a successful medication abortion. An increase in hCG concentration or a stable reading 5–15 days after using the drugs has a very high sensitivity for detecting ongoing pregnancy after medication abortion used up to 63 days. Most of the research on multi-level pregnancy tests after medication abortion has focused on the mifepristone regimen; more research is needed on using the test with misoprostol alone, especially in legally restricted settings.

Providing women comprehensive post-abortion contraceptive services, including counseling about and provision of the full range of contraceptive methods, is an important component of abortion care, including harm-reduction services. It is interesting that contraceptive uptake was similar in the telephone and in-person follow-up groups, suggesting that the addition of telephone follow-up improves contraceptive use post-abortion compared to no follow-up. All contraceptive methods other than the IUD can be offered on the first day of medication abortion, even with the mifepristone regimen [26] [27]. Provision of depoprovera and implants on the day of harm-reduction counseling may not be feasible because women may not have decided on the plans for the pregnancy and due to legal constraints.

This study has several limitations. Only about half of women invited to participate in the study agreed to participate, and we were able to contact only about half of those women for the follow-up interview. However, we observed few demographic differences between those lost to follow-up and those interviewed. Unfortunately this high loss-to-follow-up is a reality of abortion-related research in settings where the procedure is legally restricted and highly stigmatized. In addition, the findings here are specific to the model implemented in this context and may differ from other settings. For example, in other countries, such as in Central America, misoprostol is very difficult to obtain in the community, so the outcomes of the model may be different.

Worldwide, unsafe abortion is estimated to account for 8–18% of maternal deaths [2] [28] as well as a large number of medical complications [29]. Evidence from several countries indicates that liberalization of restrictive abortion laws results in improvements in health outcomes [30] [31], but the process of legal reform is often lengthy. In the interim, giving women information about evidence-based regimens of misoprostol that can be used to safely terminate an early pregnancy, as well as offering a range of follow-up options to ensure high quality post-abortion care, may reduce the harms associated with unsafe abortion. Rather than waiting for laws to change in settings where unsafe abortion is prevalent, medical professionals should explore introducing a harm-reduction model to improve women’s health in their countries.
